# Preliminary Incidence and Trends of Infections with Pathogens Transmitted Commonly Through Food — Foodborne Diseases Active Surveillance Network, 10 U.S. Sites, 2015–2018

**DOI:** 10.15585/mmwr.mm6816a2

**Published:** 2019-04-26

**Authors:** Danielle M. Tack, Ellyn P. Marder, Patricia M. Griffin, Paul R. Cieslak, John Dunn, Sharon Hurd, Elaine Scallan, Sarah Lathrop, Alison Muse, Patricia Ryan, Kirk Smith, Melissa Tobin-D’Angelo, Duc J. Vugia, Kristin G. Holt, Beverly J. Wolpert, Robert Tauxe, Aimee L. Geissler

**Affiliations:** ^1^Division of Foodborne, Waterborne, and Environmental Diseases, National Center for Emerging and Zoonotic Infectious Diseases, CDC; ^2^Oregon Health Authority; ^3^Tennessee Department of Health; ^4^Connecticut Department of Public Health; ^5^University of Colorado, Boulder, Colorado; ^6^University of New Mexico, Albuquerque, New Mexico; ^7^New York State Department of Health; ^8^Maryland Department of Health; ^9^Minnesota Department of Health; ^10^Georgia Department of Public Health; ^11^California Department of Public Health; ^12^Food Safety and Inspection Service, U.S. Department of Agriculture, Atlanta, Georgia; ^13^Center for Food Safety and Applied Nutrition, Food and Drug Administration, Silver Spring, Maryland.

Foodborne diseases represent a major health problem in the United States. The Foodborne Diseases Active Surveillance Network (FoodNet) of CDC’s Emerging Infections Program monitors cases of laboratory-diagnosed infection caused by eight pathogens transmitted commonly through food in 10 U.S. sites.* This report summarizes preliminary 2018 data and changes since 2015. During 2018, FoodNet identified 25,606 infections, 5,893 hospitalizations, and 120 deaths. The incidence of most infections is increasing, including those caused by *Campylobacter* and *Salmonella,* which might be partially attributable to the increased use of culture-independent diagnostic tests (CIDTs). The incidence of *Cyclospora* infections increased markedly compared with 2015–2017, in part related to large outbreaks associated with produce (*1*). More targeted prevention measures are needed on produce farms, food animal farms, and in meat and poultry processing establishments to make food safer and decrease human illness.

FoodNet conducts active, population-based surveillance for laboratory-diagnosed infections caused by *Campylobacter*, *Cyclospora*, *Listeria*, *Salmonella*, Shiga toxin–producing *Escherichia coli* (STEC), *Shigella*, *Vibrio*, and *Yersinia* in 10 sites covering 15% of the U.S. population (approximately 49 million persons in 2017). FoodNet is a collaboration among CDC, 10 state health departments, the U.S. Department of Agriculture’s Food Safety and Inspection Service (USDA-FSIS), and the Food and Drug Administration (FDA). Bacterial infections are defined as isolation of the bacterium from a clinical specimen or detection of pathogen antigen, nucleic acid sequences, or, for STEC,^†^ Shiga toxin or Shiga toxin genes. *Listeria* cases are defined as isolation of *L. monocytogenes* or detection of its nucleic acid sequences from a normally sterile site or from placental or fetal tissue in cases of miscarriage or stillbirth. *Cyclospora* infections are defined as detection of the parasite from a clinical specimen by direct fluorescent antibody, polymerase chain reaction, or light microscopy. Hospitalizations occurring within 7 days of specimen collection are attributed to the infection, as is the patient’s vital status at hospital discharge, or 7 days after specimen collection if the patient was not hospitalized.

Incidence per 100,000 population was calculated by dividing the number of infections in 2018 by U.S. Census estimates of the surveillance area population for 2017. A negative binomial model with 95% confidence intervals (CIs) was calculated using SAS (version 9.4; SAS Institute) to estimate changes in incidence.

Surveillance for physician-diagnosed postdiarrheal hemolytic uremic syndrome, a complication of STEC infection characterized by renal failure, thrombocytopenia, and microangiopathic anemia, is conducted through a network of nephrologists and infection preventionists and by hospital discharge data review. This report includes pediatric hemolytic uremic syndrome cases (those occurring in persons aged <18 years) identified during 2017, the most recent year for which data are available.

## Cases of Infection, Incidence, and Trends

**FIGURE 1:**
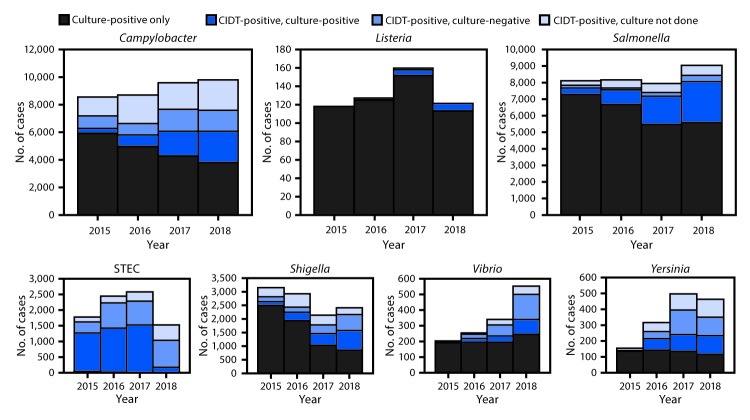
Number of infections diagnosed by culture or culture-independent diagnostic tests (CIDTs), by pathogen, year, and culture status — CDC’s Foodborne Diseases Active Surveillance Network,* 2015–2018**^†^** **Abbreviation:** STEC = Shiga toxin–producing *Escherichia coli*. * Connecticut, Georgia, Maryland, Minnesota, New Mexico, Oregon, Tennessee, and selected counties in California, Colorado, and New York. ^†^ Data for 2018 are preliminary.

**FIGURE 2:**
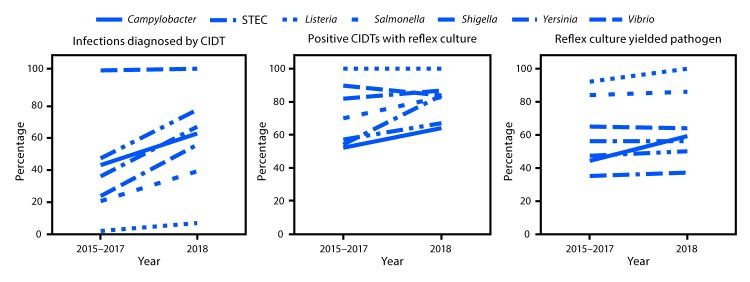
Percentage of infections diagnosed by culture-independent diagnostic tests (CIDTs), positive CIDTs with a reflex culture,* and reflex cultures that yielded the pathogen, by pathogen — CDC’s Foodborne Diseases Active Surveillance Network,**^†^** 2015–2017 and 2018**^§^** **Abbreviation:** STEC = Shiga toxin–producing *Escherichia coli*. * Culture of a specimen with a positive CIDT result. ^†^ Connecticut, Georgia, Maryland, Minnesota, New Mexico, Oregon, Tennessee, and selected counties in California, Colorado, and New York. ^§^ Data for 2018 are preliminary.

During 2018, FoodNet identified 25,606 cases of infection, 5,893 hospitalizations, and 120 deaths. The incidence of infection (per 100,000 population) was highest for *Campylobacter* (19.5) and *Salmonella* (18.3), followed by STEC (5.9), *Shigella* (4.9), *Vibrio* (1.1), *Yersinia* (0.9), *Cyclospora* (0.7), and *Listeria* (0.3) (Table). Compared with 2015–2017, the incidence significantly increased for *Cyclospora* (399%), *Vibrio* (109%), *Yersinia* (58%), STEC (26%), *Campylobacter* (12%), and *Salmonella* (9%). The number of bacterial infections diagnosed by CIDT (with or without reflex culture^§^) increased 65% in 2018 compared with the average annual number diagnosed during 2015–2017; the increase ranged from 29% for STEC to 311% for *Vibrio* (Figure 1). In 2018, the percentage of infections diagnosed by DNA-based syndrome panels was highest for *Yersinia* (68%) and *Cyclospora* (67%), followed by STEC (55%), *Vibrio* (53%), *Shigella* (48%), *Campylobacter* (43%), *Salmonella* (33%), and was lowest for *Listeria* (2%). In 2018, a reflex culture was attempted on 75% of specimens with positive CIDT results, ranging from 64% for *Campylobacter* to 100% for *Listeria* (Figure 1). The percentage of specimens with a reflex culture in 2018 was 14% higher than that during 2015–2017, ranging from a 7% decrease for STEC to a 55% increase for *Shigella* (Figure 2). Among specimens with reflex culture in 2018, the percentage that yielded the pathogen was highest for *Listeria* (100%) and *Salmonella* (86%), followed by STEC (64%), *Campylobacter* (59%), *Shigella* (56%), *Yersinia* (50%), and *Vibrio* (37%) (Figure 1) (Figure 2).

**TABLE:** Number of cases, hospitalizations, and deaths caused by bacterial and parasitic infections, incidence rate, and percentage change compared with 2015–2017 average annual incidence rate, by pathogen — CDC’s Foodborne Diseases Active Surveillance Network,* 2018^†^

Pathogen	2018				2018 compared with 2015–2017
	No. of cases	No. (%) of hospitalizations	No. (%) of deaths	IR^§^	% (95% CI) Change in IR^¶^
**Bacteria**					
*Campylobacter*	9,723	1,811 (18)	30 (0.3)	19.6	12 (4 to 20)
*Salmonella*	9,084	2,416 (27)	36 (0.4)	18.3	9 (3 to 16)
Shiga toxin–producing Escherichia coli**	2,925	648 (22)	13 (0.4)	5.9	26 (7 to 48)
*Shigella*	2,414	632 (26)	1 (0.04)	4.9	−2 (−24 to 26)
*Vibrio*	537	151 (28)	9 (2)	1.1	109 (72 to 154)
*Yersinia*	465	95 (20)	4 (0.9)	0.9	58 (26 to 99)
*Listeria*	126	121 (96)	26 (21)	0.3	−4 (−23 to 21)
**Parasite**					
*Cyclospora*	332	19 (5)	1 (0.3)	0.7	399 (202 to 725)
**Total**	**25,606**	**5,893 (23)**	**120 (0.5)**	—	—

**Abbreviation:** CI = confidence interval; IR = incidence rate.

* Connecticut, Georgia, Maryland, Minnesota, New Mexico, Oregon, Tennessee, and selected counties in California, Colorado, and New York.

^†^ Data are preliminary.

^§^ Per 100,000 population.

^¶^ Increase or decrease.

** All serogroups were combined because it is not possible to distinguish among them using culture-independent diagnostic tests.

Among 7,013 (87%) serotyped *Salmonella* isolates, the three most common were Enteritidis (2.6 per 100,000 population), Newport (1.6), and Typhimurium (1.5), similar to those during 2015–2017. Among 1,570 STEC isolates tested, 440 (28%) were determined to be O157. Among 662 non-O157 STEC isolates serogrouped, the most common were O103 (31%), O26 (28%), and O111 (24%). The incidence compared with 2015–2017 remained unchanged for both O157 and non-O157 STEC.

FoodNet identified 54 cases of postdiarrheal hemolytic uremic syndrome in children (0.49 cases per 100,000) during 2017; 36 (67%) occurred among children aged <5 years (1.22 cases per 100,000). Incidence was not significantly different compared with that during 2014–2016.

## Discussion

*Campylobacter* has been the most commonly identified infection in FoodNet since 2013. It causes diarrhea, sometimes bloody, and 18% of persons are hospitalized. A rare outcome of *Campylobacter* infection is Guillain-Barré syndrome, a type of autoimmune-mediated paralysis. Poultry is a major source of *Campylobacter* (*2*). In August 2018, FSIS began using a new testing method; in a study of that method, *Campylobacter* was isolated from 18% of chicken carcasses and 16% of chicken parts sampled (*3*). FSIS currently makes aggregated test results available and intends to update performance standards for *Campylobacter* contamination.

The incidence of infections with Enteritidis, the most common *Salmonella* serotype, has not declined in over 10 years. Enteritidis is adapted to live in poultry, and eggs are an important source of infection (*4*). By 2012, FDA had implemented the Egg Safety Rule,^¶^ which requires preventive measures during the production of eggs in poultry houses and requires subsequent refrigeration during storage and transportation, for all farms with ≥3,000 hens. In 2018, a multistate outbreak of Enteritidis infections was traced to eggs from a farm that had not implemented the required egg safety measures after its size reached ≥3,000 hens (*5*). Chicken meat is also an important source of Enteritidis infections (*4*). In December 2018, FSIS reported that 22% of establishments that produce chicken parts failed to meet the *Salmonella* performance standard (USDA-FSIS *Salmonella* verification testing program**). The percentage of samples of chicken meat and intestinal contents that yielded Enteritidis were similar in 2018 to those during 2015–2017 (USDA-FSIS, unpublished data). In contrast, a decline in serotype Typhimurium isolated from the same sources was observed during the same period. This trend coincides with declines in Typhimurium human illnesses. Changes in poultry production practices, including vaccination against Typhimurium, might have resulted in these declines (*6*). In the United Kingdom, vaccination of both broiler and layer chickens against Enteritidis, along with improved hygiene, was followed by a marked decrease in human Enteritidis infections (*7*).

Produce is a major source of foodborne illnesses (*2*). During 2018, romaine lettuce was linked to two multistate outbreaks of STEC O157 infections (*8*). The marked increase in reported *Cyclospora* infections was likely attributable to several factors including produce outbreaks and continued adoption of DNA-based syndrome panel tests (*1*). Improved agricultural practices are needed to prevent produce-associated infections. FDA provides technical assistance to task forces created by the produce industry, to determine how to prevent contamination of romaine lettuce and facilitate outbreak investigations by improving product labeling and traceability. In 2018, FDA expanded surveillance sampling of foreign and domestically grown produce to assess its safety (*9*). FDA is implementing the Produce Safety Rule,^††^ with routine inspections of large produce farms planned this spring. Because produce is a major component of a healthy diet and is often consumed raw, making it safer is important for improving human health (*10*).

The findings in this report are subject to at least three limitations. First, the changing diagnostic landscape makes interpretation of incidence and trends more complex. Increases in reported incidence might be attributable entirely, or in part, to changes in clinician ordering practices, increased use of DNA-based syndrome panels that identify pathogens not routinely captured by traditional methods, and changes in laboratory practices in response to the availability of these panels. Second, some CIDT results might be false positives. Finally, year-to-year variations, attributable in part to large outbreaks, might not indicate sustained trends.

The need to obtain and subtype isolates from ill persons is becoming an increasing burden to state health departments but is critical for maintaining surveillance to detect and investigate outbreaks, evaluating prevention efforts, and developing targeted control measures. Measures that might decrease foodborne illnesses include enhanced efforts targeting *Campylobacter* contamination of chicken; strengthening prevention measures during egg production, especially within small flocks; vaccinating poultry against *Salmonella* serotype Enteritidis; decreasing *Salmonella* contamination of produce, poultry, and meat; and continued implementation of the Food Safety Modernization Act, specifically FDA’s Produce Safety Rule. FoodNet continues to collect data and develop analytic tools to adjust for changes in diagnostic testing practices and test characteristics. These actions, along with FoodNet’s robust surveillance, provide data to help evaluate the effectiveness of prevention efforts and determine when additional measures are needed.

**Summary**

**What is already known about this topic?**

The incidence of foodborne infections has remained largely unchanged. Clinical laboratories are increasingly using culture-independent diagnostic tests (CIDTs) to detect enteric infections. CIDTs benefit public health surveillance by identifying pathogens not routinely detected by previous methods but complicate data interpretation.

**What is added by this report?**

The incidence of most infections increased during 2018 compared with 2015–2017; this might be partially attributable to increased CIDT use. The incidence of *Cyclospora* infections increased markedly, in part related to large outbreaks associated with produce. The number of human infections caused by *Campylobacter* and *Salmonella*, especially serotype Enteritidis, remains high.

**What are the implications for public health practice?**

As use of CIDTs increases, it is important to obtain and subtype isolates and interview ill persons to monitor prevention efforts and develop more targeted prevention and control measures to make food safer and decrease human illness.

## Footnotes

* Connecticut, Georgia, Maryland, Minnesota, New Mexico, Oregon, Tennessee, and selected counties in California, Colorado, and New York (https://www.cdc.gov/foodnet).

† STEC cases are defined as identification of Shiga toxin or its genes by any laboratory; it is not possible to distinguish among serogroups using CIDTs.

§ Culture of a specimen with a positive CIDT result.

¶
https://www.fda.gov/Food/GuidanceRegulation/GuidanceDocumentsRegulatoryInformation/Eggs/ucm170615.htm .

**
https://www.fsis.usda.gov/wps/portal/fsis/topics/data-collection-and-reports/microbiology/salmonella-verification-testing-program .

††
https://www.fda.gov/Food/GuidanceRegulation/FSMA/ucm334114.htm .
