# *Erratum:* Vol. 68, No. 16

**DOI:** 10.15585/mmwr.mm6820a5

**Published:** 2019-05-24

In the report “Preliminary Incidence and Trends of Infections with Pathogens Transmitted Commonly Through Food — Foodborne Diseases Active Surveillance Network, 10 U.S. Sites, 2015–2018,” in the figure on page 371 (Figure 1), for Shiga toxin–producing *Escherichia coli* (STEC), the value for 2018 culture-independent diagnostic test (CIDT)–positive, culture-positive cases should have been **1,559**, resulting in a total of **2,925** STEC cases in 2018, as seen in the Table on page 370.

**FIGURE 1:**
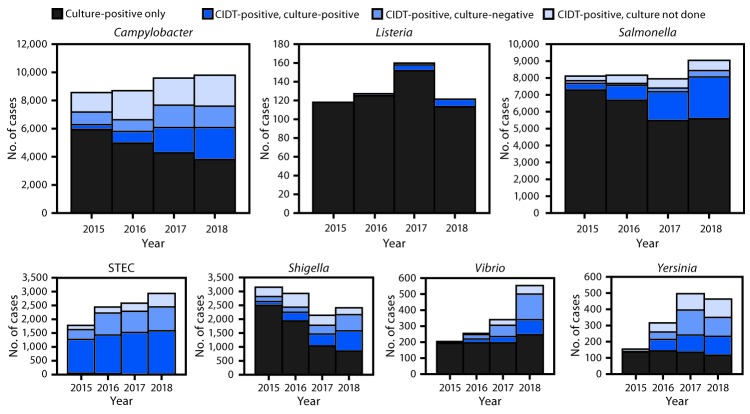
Number of infections diagnosed by culture or culture-independent diagnostic tests (CIDTs), by pathogen, year, and culture status — CDC’s Foodborne Diseases Active Surveillance Network,* 2015–2018^†^ **Abbreviation: **STEC = Shiga toxin–producing *Escherichia coli*. * Connecticut, Georgia, Maryland, Minnesota, New Mexico, Oregon, Tennessee, and selected counties in California, Colorado, and New York. ^^†^^ Data for 2018 are preliminary.

